# [Corrigendum] Crocetin induces apoptosis of BGC‑823 human gastric cancer cells

**DOI:** 10.3892/mmr.2023.13038

**Published:** 2023-06-21

**Authors:** Ke He, Picheng Si, Hanning Wang, Usman Tahir, Kaiyun Chen, Jinfeng Xiao, Xiaopeng Duan, Rui Huang, Guoan Xiang

Mol Med Rep 9: 521–526, 2014; DOI: 10.3892/mmr.2013.1851

Following the publication of the above article, an interested reader drew to the authors’ attention that Fig. 2 (showing morphological characteristics of cultured BGC-823 cells as visualized by microscopic analysis) and Fig. 3 (showing crocetin-induced apoptosis of the BGC-823 cells) on p. 523 appeared to feature panels containing overlapping data. The authors re-examined their original data, and realized that inadvertent errors were made during the compilation of these figures; specifically, the data shown in Fig. 2C (for the the 5-μM docetaxel group) and Fig. 3D (for the DMSO group) were selected incorrectly.

The corrected versions of Figs. 2 and 3 are shown below and on the next page, now featuring the correct data for Figs. 2C and 3D. All the authors agree with the publication of this corrigendum, and are grateful of the Editor of *Molecular Medicine Reports* for granting them the opportunity to publish this. Furthermore, they regret that these errors were introduced into the paper, even though they did not substantially alter any of the major conclusions reported in the paper, and apologize to the readership for any inconvenience caused.

## Figures and Tables

**Figure 2.**
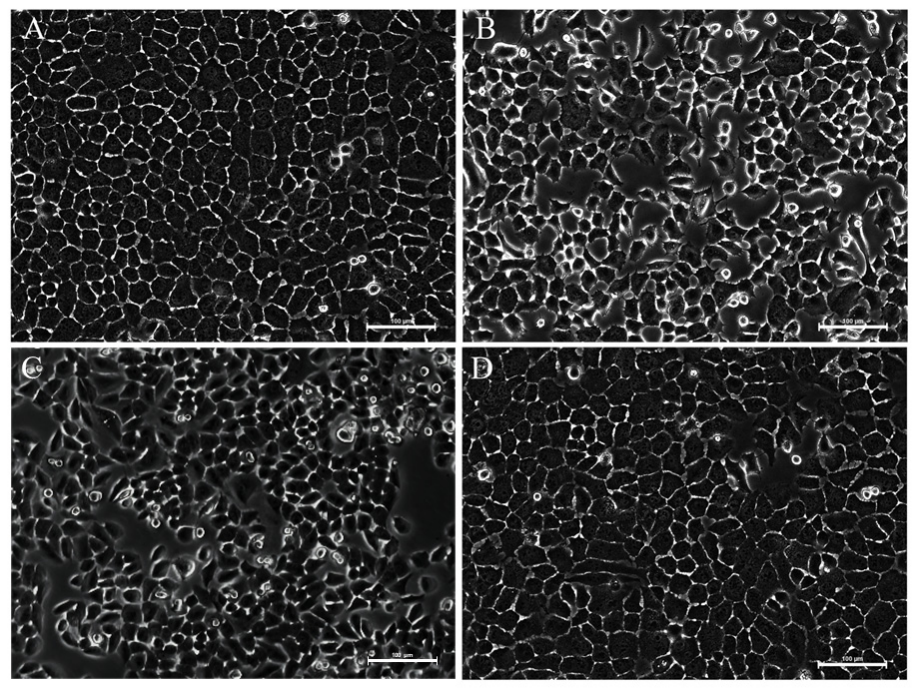
Morphological characteristics of cultured BGC-823 cells as visualized by microscopic analysis. (A) The control group, (B) the 200-μM crocetin group, (C) the 5-μM docetaxel group and (D) the DMSO group (scale bar, 100 μm).

**Figure 3.**
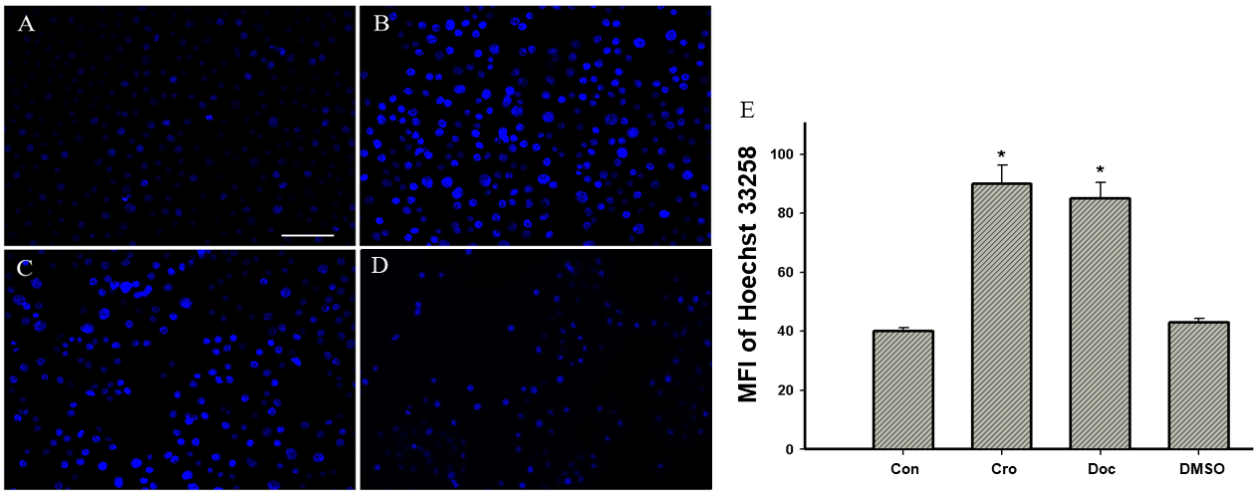
Crocetin-induced apoptosis in BGC-823 cells. Apoptotic nuclei were visualized by Hoechst 33258 staining. (A) Control (Con), (B) crocetin (Cro), (C) docetaxel (Doc) and (D) dimethylsulfoxide (DMSO) groups (scale bar, 100 μm), and (E) quantitative analysis of the fluorescence intensity (*P<0.01 vs. the control group). MFI, mean fluorescence intensity.

